# Nutritional Intake and Dietary Knowledge of Athletes: A Scoping Review

**DOI:** 10.3390/nu17020207

**Published:** 2025-01-07

**Authors:** Caroline Hopper, Elaine Mooney, Amanda Mc Cloat

**Affiliations:** National Centre for Excellence in Home Economics, ATU-St Angelas, F91 C634 Sligo, Ireland; elaine.mooney@atu.ie (E.M.); amanda.mccloat@atu.ie (A.M.C.)

**Keywords:** sports nutrition, athletes’ nutritional knowledge, dietary intake of athletes, nutrition education, food skills, nutrition and diet

## Abstract

**Background**: Sports nutrition is a rapidly developing field of study, and optimum nutrition can have a crucial impact on athletic performance and, in turn, overall well-being. Nutritional knowledge and dietary intake of athletes are paramount in terms of reaching optimum athletic performance and promoting recovery. This review will scope the current literature in relation to nutritional knowledge and dietary intake of athletes in order to establish gaps in the research that require further exploration. **Methods**: A review of papers (*n* = 21) related to athletes’ nutritional knowledge and dietary intake in Sage, Pub-Mud, EBESCO and Sports-Discus databases was undertaken up to October 2024. Each of these papers met the strict eligibility criteria for inclusion following the adoption of the guidelines of Preferred Reporting Items for Systematic Reviews and Meta-analysis Extension for Scoping Reviews (PRISMA–ScR). **Results**: Studies reported that gaps exist in the nutritional knowledge and awareness of athletes in relation to nutrient intake and the recommended dietary guidelines. Furthermore, a disparity in knowledge exists with females overall exhibiting superior awarenesses in comparison to male counterparts. The findings of this study suggest that food supplements are being used to compensate for a shortfall in nutrition. A lack of access to evidence-based nutritional advice and practical, hands-on nutritional education have been cited as major barriers to enhancing and addressing nutritional awareness and knowledge. **Conclusions**: This scoping review mapped the existing literature relating to athletes’ nutritional knowledge and dietary practices and, in turn, revealed critical gaps and barriers The review established the need for further research to explore and address these gaps.

## 1. Introduction

Sports nutrition is the study and application of how to use nutrition to support all areas of athletic performance [1]. This encompasses both nutritional knowledge and dietary intake to promote optimum performance. Peak physical performance is the optimum goal for athletes. Understanding the connection between diet and performance is essential for developing well-informed and targeted sports nutrition strategies [2]. Optimum dietary intake and nutritional knowledge have been cited as key factors influencing an improvement in the athletic performance and health status of athletes [3,4]. Diet and nutrition play a foundational role in supporting athletes’ health, training and, in turn, competition goals. Whether an elite athlete or a recreational participant, it is important to have a good understanding of nutritional science. This can be the differentiating factor between good and exceptional performance [5]. As the competitive expectation rises, so too does the need for evidence-based nutritional strategies that can support players in achieving peak performance.

For athletes, whose training and schedules can be demanding, the importance of a well-balanced and strategic diet cannot be overstated [6]. However, despite the growing acknowledgement for the role of nutrition in sport, there is limited literature specifically addressing the gaps in nutritional knowledge, dietary needs and practices of athletes. Given the direct impact of nutrition on athletic performance, recovery and indeed overall health, a deeper understanding of the specific dietary practices and knowledge gaps of athletes is crucial. Despite increasing attention to sports nutrition, there remains a lack of comprehensive reviews that map existing evidence and identify targeted opportunities for improvement in this area.

The aim of this scoping review is to investigate and collate the existing evidence on the relationship between nutritional knowledge and dietary intake among athletes in order to establish gaps in the research that require further exploration. This review seeks to contribute to the evolving field of sports nutrition by reporting on the breadth of the existing literature whilst offering insights to contribute to athletes, coaches and nutritionists, in turn, optimizing dietary strategies to promote enhanced performance and recovery.

## 2. Materials and Methods

A scoping review was selected as the most appropriate method for this investigation, as it is a type of research synthesis that can map the existing literature of a topic in a bid to identify the key concepts, highlight gaps in the literature, and collate types of evidence [7]. Due to the heterogeneous nature of the included studies and the exploratory nature of the study aim, a meta-analysis approach was not appropriate; hence, the results of this study were narratively synthesized.

**Figure 1:**
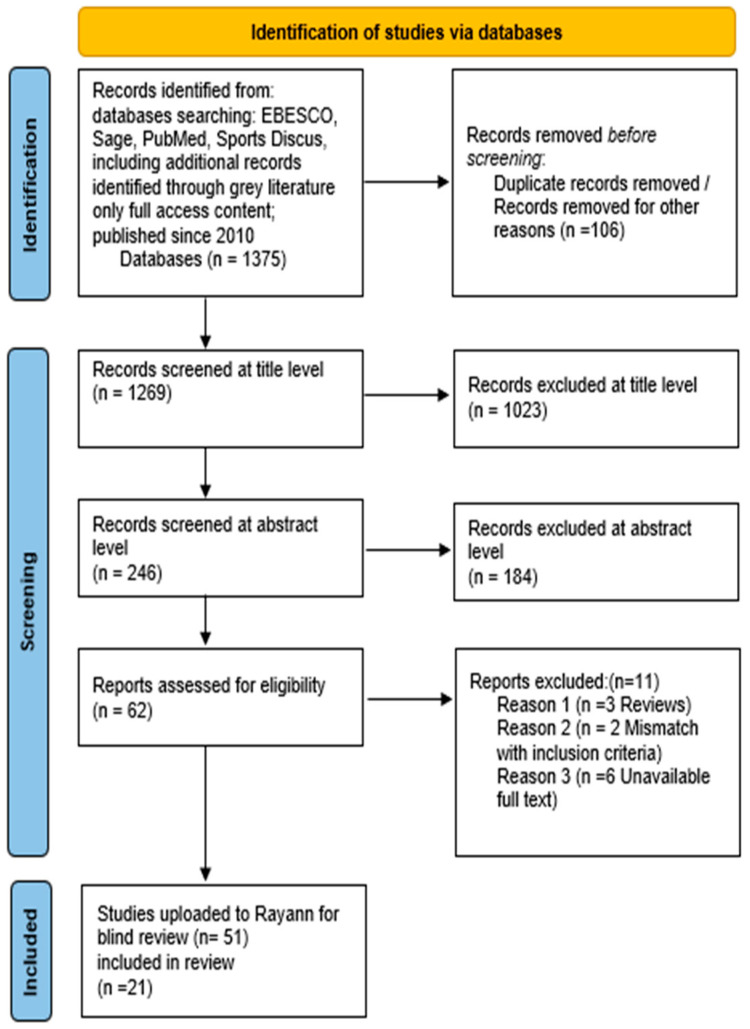
Preferred reporting items for Systematic Reviews and Meta-Analyses (PRISMA) Flow Diagram of Abstract and Full Text Screening.

This scoping review follows the guidelines published by the Preferred Reporting Items for Systematic reviews and Meta-Analyses (PRISMA) extension for Scoping Reviews (PRISMA-ScR) with reference to Arskey and O’ Malley’s [8] original framework (2005) (Figure 1), later developed by Levac et al. [9]. This scoping review adhered to the PRISMA-ScR (Preferred Reporting Items for Systematic Reviews and Meta-Analyses extension for Scoping Reviews) guidelines. This ensured full transparency and methodological rigor throughout the engagement of both the review and study selection process.

### 2.1. Inclusion Criteria

The search was limited to open access articles, published in English since 2010. As research in the sphere of nutrition, sports science, and dietary education is a rapidly evolving field, all of the included articles have been published since 2010. This restriction allows for only the inclusion of evidence that reflects current practices, knowledge and information that is up to date and relevant. Table 1 depicts studies based on year of publication. The search parameters were then further refined to exclude any studies that included minors, non-athletes and non-peer-reviewed articles. Studies included participants over the age of 18. A diverse level of competitiveness was included; this ranged from recreational to elite athletes, and a range of sporting disciplines were included. These strict criteria ensured the inclusion of high-quality, applicable literature with a specific focus on the study population and concept.

### 2.2. Exclusion Criteria

The exclusion criteria for this study applied to studies that involved minors (under 18 years of age), or non-athlete populations, or whether the studies were not peer-reviewed. This ensured there was relevance and rigor to the studies included. The specifics of the inclusion and exclusion criteria allowed the author to narrow down the search to include studies that specifically addressed the inter-relationship between performance and dietary knowledge of adult athletes within a peer-reviewed context.

### 2.3. Search Strategy and Database Selection

A systematic search strategy was developed and conducted to identify relevant sources related to the conceptual model and the developed PCC (Population, Concept, Context) framework. This framework defined the population for inclusion as athletes and included a diverse range of age groups whilst excluding minors. A diverse level of competitiveness was included encompassing athletes ranging from recreational to elite. A range of sporting disciplines were included. The underpinning concept focused on nutritional knowledge. This can be defined as an understanding of dietary guidelines, nutrient needs, and practical application, in turn influencing one’s health [10]. The context of this review encompassed a range of environments, for example, where athletes engage in training, competition and recovery. This, in turn, will highlight factors that influence nutritional behaviors. Key words were extracted and selected from the initial research question, aim and objectives of this study. The search strategy had a specific focus on papers related to athletes’ nutritional knowledge, dietary intake and players’ performance and recovery with reference to sports nutrition. The aim of this strategy was to comprehensively capture indicators of the relationship between sports nutrition science, performance, nutrition knowledge and dietary intake within the context of sport.

### 2.4. Keywords and Search Terms

The keywords and search terms were carefully selected to ensure the retrieval of relevant literature. The initial search terms included “sports nutrition” and “athletes’ nutritional knowledge” and “Dietary Intake”. These terms are directly aligned to and target the specific athlete population under study. These terms were then combined with specific inter-related keywords including “Performance”, and terms that are directly related to dietary knowledge and food literacy in sports, including “food literacy in sport”, “nutrition education”, “nutrition”, and “diet”. In addition to these terms, further key words including “food” and “food intake” were utilized to capture further studies with a focus on dietary behaviors of the study population. See Table 1 for the inter-relationship of search terms derived from each concept.

### 2.5. Boolean Operators

To further refine the search, Boolean operators (AND, OR) were used to combine keywords. The terms “Sports nutrition” and “athletes’ nutritional knowledge” were subsequently paired with the terms “Performance” AND “nutrition education” AND “food literacy in sport”, AND “nutrition” OR “diet”, thus ensuring that the search results incorporated a broad range of relevant studies.

### 2.6. Study Selection

Following the framework of Arskey and O Malley [8], databases including Pub-med, Sports Discus, Sage, EBESCO along with gray literature were screened for papers matching the initial search terms and criteria. These databases were included due to their relevance to sports science, nutrition, and health research. Other databases were excluded due to limited coverage of the key concepts. There was also consideration of gray literature. This included reports and informal studies. This aided in capturing a full picture of the pertinent research within this area and allowed for a more comprehensive mapping of the topic. The initial search retrieved a total of 1375 articles from the peer-reviewed literature. A comprehensive literature retrieval such as this was essential to ensure that no relevant studies were overlooked in the initial screening stages. Following the removal of duplicate records (*n* = 106), the remaining articles (*n* = 1269) underwent a further screening process. This screening was conducted at both title and abstract levels to assess the relevance of each study to the research focus. Articles passed initial screening if their title contained words or sentences that were included in the search terms derived from the underpinning concepts (Table 1). After this detailed screening process, eligible studies were then uploaded to referencing management software Zotero 6.0.26. The remaining articles (*n* = 51) were then exported to Rayyan screening software, https://new.rayyan.ai/ (accessed on 9 November 2024). Rayyan is a software used for research collaboration. Rayyan was used to promote rigor in the search strategy, and to facilitate and streamline the selection of eligible papers. Two reviewers (E.M., C.H.) were consistently involved and independently blind-screened all article titles and abstracts against the inclusion and exclusion criteria. Conflicts between reviewers were resolved by a third reviewer (A.M.C.). The reference lists of eligible articles were then searched for identification of the relevant literature for inclusion. Each of the included studies met all the criteria and were found to be directly relevant to the nutritional knowledge and dietary intake of athletes within the context of the PCC framework. Following Rayyan screening and selection, 21 papers were deemed suitable for inclusion in this scoping review. A basic appraisal of the quality of each included study was conducted to identify any potential biases and, in turn, assess the reliability of the findings presented.

### 2.7. Data Charting and Synthesis

The final selection of 21 studies provided a robust foundation for the scoping review, offering insights into the relationship between nutritional knowledge and dietary intake of athletes. The process of screening to include the selected studies is depicted in Figure 1. For each included study, the author, year of publication, country, study aims, population, sample size, methods, key findings and conclusion were mapped to depict relevance of the findings (Table 2).

## 3. Results and Discussion

**Figure 2:**
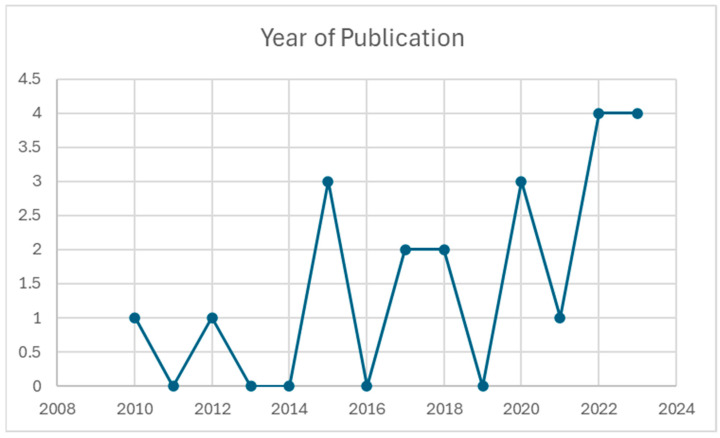
Publication by Year Frequency (*n* = 21).

As depicted in Figure 2, research in this area has increased over the years with 57% (*n* = 12) of the studies included in this review conducted since 2020.

Following the screening of papers, a total of twenty-one studies were deemed eligible for inclusion in this review. As outlined in Table 1, all these studies were published since 2010 and represent a diverse range of countries: Ireland, USA, Pakistan, Finland, Spain, Iran, and Australia. Each study met the specific inclusion criteria established for this review.

Regarding the research methods of the included studies, two used qualitative methods, thirteen used quantitative methods, and six used a mixed-methods approach. Five of the quantitative studies used validated tools. There was greater participation noted from male respondents under study. While twelve of the twenty-one included studies that recruited both male and female participants, the overall number of participants were male. Only one study solely focused on female participants, while eight studies solely focused on male participants.

The study outcome and results were mapped according to common findings. The synthesis of the findings was guided by an iterative, narrative-driven process to allow for identification and organization of the key findings across the studies. The findings from the scoping of the literature revealed several gender differences; for example, female athletes demonstrated greater efficacy in hands-on practical applications and food-related skills, while male athletes were more likely to rely on dietary supplementation to enhance their performance [24]. Females also demonstrated superior nutritional knowledge in comparison to male counterparts [16].

Several significant barriers to healthy eating and food choices were identified; for example, time constraints, lack of nutritional knowledge, and cost emerged as key factors that influence athletes’ dietary habits [20,21,26]. Analysis of the pertinent literature in relation to the interconnection of nutritional knowledge and dietary behaviors of athletes yielded five key findings. The findings have been organized into five key areas: nutritional knowledge and awareness, dietary practices and food intake, impact of nutrition on performance, nutritional educational programs, and challenges and barriers to optimal nutrition.

### 3.1. Nutritional Knowledge and Awareness

Fourteen of the twenty-one included studies within this scoping review highlighted a generally low to moderate level of nutritional knowledge among athletes. Mc Crink et al. [6] conducted a study on the nutritional awareness of Irish Gaelic football players. The findings of this study indicated that despite their status as competitive athletes, many of the study cohort had poor to average nutritional knowledge and lacked a comprehensive understanding of nutrient requirements, timing of nutrient intake, and the role of hydration in athletic performance with 19% of these athletes being dehydrated prior to exercise and exhibiting prevalent nutrient deficits [6]. The substandard nutritional knowledge was also echoed in a study by Mitchell et al. [13] with no significant differences in dietary knowledge observed between elite and amateur players. These findings were further reinforced in a study by O’Cathain et al. [14] with Gaelic football players failing to meet sports nutrition guidelines. Within an international context, similar practices are mirrored; a study by Trackman et al. [5] investigated Australian football athletes’ (elite and amateur) lack of awareness of current sport nutrition guidelines. The findings indicated that knowledge of macronutrients, weight management and alcohol was more advanced than knowledge of supplementation, micronutrients and key sports nutrition guidelines [5]. These findings within the Australian context remain consistent with the Irish cohort. It was further suggested that the gap in knowledge and awareness often led to suboptimal dietary intake that could hinder performance. However, there was some evidence that on “game day”, purposeful nutritional planning was evident [14]. Murphy and O Reilly [12] investigated the association between nutritional knowledge and dietary intake amongst a sample of Irish male hurling players, also falling under the umbrella of Gaelic football games. Nutritional knowledge was measured using a validated Sports Nutritional Knowledge Questionnaire (SNKQ). A total of 266 participants ranging from amateur to elite were selected for inclusion. Again, nutritional and energy intake deficits were reported, with the intentional limit of carbohydrates to reduce body fat negatively impacting performance. Avenues used by athletes to acquire information were also noted as a cause for concern [4]. Malsagova et al. [27] described how dietary accessibility, social support, habits, and, in particular, marketing can influence dietary behaviors. There is also a growing phenomenon of social media influencers promoting products to enhance performance. However, the overall impact of these factors is unclear, and further research in this area is required.

Malsagova et al. [27] further discuss how nutrition is considered to be the cornerstone of athletic performance and highlight the importance of adequate consideration of post-workout nutritional recommendations as being fundamental to the effectiveness of the recovery and adaptive processes. This statement contrasts with the awareness of nutritional guidelines and dietary intake of the athlete population in relation to nutrient recommendations. One study in particular, by Boucherville et al. [26], evaluated the nutritional knowledge of recreational athletes. Findings of this study indicated that significant gaps exist in participants’ knowledge of sports nutrition. This is also reflected in a study by Trackman et al. [5] where athletes demonstrated proficiency in knowledge of macronutrients, weight management and alcohol, superior to knowledge of supplementation. Non-elite respondents demonstrated greater proficiency and higher scores than elite players. These findings remain consistent across the literature. The gaps are particularly prevalent in relation to knowledge of nutritional recommendations for the macronutrients protein and carbohydrate. A majority of the participants in this study demonstrated proficiency in knowledge of post-workout protein requirements; however, less than half of the participants (40.8%) could correctly identify the recommended daily protein intake for athletes [26]. These findings further indicate that knowledge of the impact of carbohydrate requirements during exercise was better understood than protein, with 63.8% of participants knowledgeable of the impact of carbohydrates in relation to energy requirements and its role in stabilizing blood sugar levels; however, in contrast, a minority of participants (37.9%) demonstrated awareness of the recommended nutritional intake of carbohydrates during exercise. This study also highlighted a gap in hydration knowledge and practices. A minority of participants (10.5%) demonstrated proficient knowledge of the role of hydration in maintaining adequate blood volume during training. In general, these findings further highlight the need for further education in sports nutrition specifically tailored to address this gap. This is also seen on an international scale as demonstrated in a study by Jessri [17]. This particular study was conducted with Iranian basketball and football players. The findings of this study indicated that female participants demonstrated greater proficiency than males, and there was a clear need for evidence-based nutritional guidance. Parks et al. [21] describe that as athletes face nutritional barriers, there is an inconsistency with the source of information and that perhaps a collaborative approach between the trainer/coach and dietician may be a mode to enhance the delivery of evidence-based nutritional knowledge. It was also noted in a study by [23] that there is a need for more targeted nutritional educational plans encompassing a multi-disciplinary approach.

Education surrounding the significance of carbohydrate intake and its importance in supporting training and performance is warranted amongst athletes [28]. Findings further indicated that protein intake was generally met or, in some cases, exceeded recommended intake, which supports Trackman’s [5] findings. In general, the recorded suboptimal dietary practices of athletes strengthens the argument for improved, tailored nutritional knowledge and awareness to promote optimum performance and health.

A key concept within this review, and recurrent in the findings, is that of good nutritional intake and what it constitutes. Good nutritional intake of athletes encompasses a diet that meets an athlete’s specific energy, macronutrient, and micronutrient requirements to support optimal performance, recovery, and overall health [29]. While the needs of athletes can vary based on a variety of factors including sporting field, training intensity and individual physiology, mainstream dietary guidelines can offer a general framework for balanced nutrition. Interpretations of a what a good diet is may differ widely due to cultural influences, individual dietary philosophies, and exposure to emerging dietary trends.

### 3.2. Dietary Practices and Food Intake

This scoping review found that there was a varied approach to the dietary practices of athletes. Several common threads were highlighted including insufficient carbohydrate intake and inadequate pre- and post-training nutrition. Mc Daid et al. [30] reported the role of the commercial food industry and the reliance on supplementation. Findings of this study indicated that the routine use of food supplements was reported by approximately 60% of the study cohort. According to the European Parliament [31], a food supplement is used to complement the diet causing a concentrated nutritional or physiological effect. It can be found in various forms including tablet, pastilles, powder sachet or liquid [31]. It is designed to be consumed in small unit quantities.

The use of supplementary nutrition suggests that players might be compensating for dietary gaps with products rather than whole foods [30]. A study by Sánchez-Oliver et al. [24] explores the effects of supplementation on the athletic performance of rugby players. The study focused on the role of supplementation in enhancing energy availability, recovery and, in turn, overall health. It highlighted the efficacy of specific supplements (protein and creatine) in supporting muscle repair and improving strength and endurance. The most common motivation for this supplementation was enhanced performance. The findings emphasize the importance of sound evidence-based supplementation practices to maximize benefits and minimize potential risks for athletes as some of these products are purchased online. Mc Daid et al. [30] state that the most frequently reported types of food supplements consumed are those of protein, Vitamin D and Vitamin C with each popular amongst both elite and amateur athletes. Further to this, a study by Duggan, Collins and Keane [32] investigated the dietary intake of female Irish Gaelic games athletes. This study reported suboptimal intakes of key micronutrients amongst this cohort. This reiterates the need for further research in this area and a targeted approach to address this gap in knowledge and practice. Jenner et al. [33] indicates that a targeted nutritional intervention strategy could be a practical method of improving dietary intake, in turn benefiting a broad range of athletes within differing sporting disciplines, who are currently failing to meet nutritional recommendations.

### 3.3. Impact of Nutrition on Performance

There is well-established evidence that there is a direct correlation between dietary intake, performance and recovery in field-based team sports including soccer and rugby. O Caitlin et al. [14] conducted a study to determine the dietary intake of Irish Gaelic games athletes, and findings indicated that regardless of playing level, players consumed less than the recommended level of carbohydrates to support optimal performance and recovery; however, results showed that protein and fat were consumed in line with general sport nutrition guidelines. Mc Crink et al. [6] examined the relationship between dietary intake and performance outcomes. In contrast to the reported macronutrient deficits, diets high in carbohydrate were consistently associated with improved energy, performance, endurance and reduced levels of fatigue during matches. This was also noted by Mc Guire [28], where exercise energy expenditure was greater that energy availability. Surprisingly, in this study, Gaelic football players failed to meet the recommended energy intake levels, and therefore, there is an increased risk of low energy availability and decreased performance. This particular study examined energy availability in 20 elite male Gaelic football players. These findings are consistent with a study conducted by Werner et al. [16] who assessed the nutritional knowledge of college athletes. Findings of this study indicated that athletes had lower nutritional knowledge. This, in turn, increased the athletes’ risk factor for decreased athletic performance and increased risk of injury.

Adequate protein intake was reported, and this could be directly linked to faster recovery times and better muscle maintenance. Supplementation of protein was also widely reported. Evidence exists to support the consequences of poor hydration strategies [34] as cited in Duggan et al. [32]. The results of that study found that 25% of female athletes were recorded as dehydrated pre- and post-exercise, much lower than their male counterparts that reported 35% and 48%, respectively. Further to this, the ill effects of dehydration on performance can include an increase in core body temperature, cardiovascular strain, and impaired cognitive performance. Proper hydration has been shown to positively impact cognitive function, decision-making, and overall performance.

Limited understanding and application of sports nutrition can contribute to a macronutrient deficit. This, in turn, can lead to increased fatigue, a greater risk of injury, and prolonged recovery. Furthermore, inadequate dietary practices can also result in long-term health consequences, some of which include chronic energy deficits and metabolic disruptions. This further highlights the need for sound nutritional knowledge and dietary practices to promote performance and recovery and, in turn, strengthens the argument for the need of targeted nutritional interventions.

### 3.4. Nutritional Educational Programs

The need for targeted nutrition education programs, specifically tailored to meet the needs of athletes due to poor dietary behaviors and lack of nutritional knowledge, has been acknowledged [35]. A study by Lydon et al. [15] aimed to assess the impact of a food skills and nutrition education workshop, with young Gaelic football players, on cooking skill efficacy, food literacy and willingness to adopt positive dietary practices. The study highlighted that many athletes struggle with the practical application of knowledge due to insufficient skills. It also showcased a gender disparity with females exhibiting greater confidence with practical application. The effectiveness of a tailored nutritional intervention was proven to effectively improve nutritional knowledge, cooking skills and application of nutritional guidelines. Studies that implemented educational models reported more positive outcomes, including increased carbohydrate intake, improved hydration strategies, and better overall dietary quality [25]. Renard et al. [11] conducted a study to assess the inter-relationship between cooking and food skills confidence among 266 team sport athletes. Results indicated that females had higher confidence levels in both cooking and food skills. This is also echoed by Murphy and O’Reilly [12]. The results showed that factors including gender, culinary training, health interest and food engagement had a significant bearing on confidence levels. These results suggest that male athletes may benefit from targeted educational interventions to improve these skills [11,19]. These findings suggest that targeted nutrition education can effectively motivate behavior change and support athletic performance. This further highlights the need for further exploration of the motivational factors for enhanced dietary practices. Participation in meal preparation has been associated with improved eating behaviors, and the benefits have been showcased with the lasting impact carrying into adulthood [36]. Further to this, only 14.2% of one study cohort reported having previously accessed nutrition education, and this was primarily at the elite level [13].

### 3.5. Challenges and Barriers for Athletes in Accessing and Maintaining Optimum Nutrition

This scoping review identified a number of barriers and challenges faced by athletes in accessing and maintaining optimal nutrition. These challenges included a lack of access to professional nutritional advice, lack of baseline nutrition knowledge assessment, lack of evidence-based knowledge on supplementation and time constraints due to balancing sports, work, and other commitments [5,25,37]. Additionally, cultural factors and personal dietary preferences were found to influence dietary choices, sometimes leading to suboptimal nutritional practices [22,38]. A study by Brauman [20] explored the barriers faced by college-level athletes when it comes to maintaining a healthy diet. The main significant challenges isolated were lack of time, easy access to unhealthy foods, cost of healthy foods, and lack of nutritional knowledge and cooking skills. “Lack of time” was commonly cited and therefore was the top barrier. This theme reflected the athletes’ busy schedules with classes, training, and social commitments. This aligns with findings from Beasley [37]. Additionally, the cost of healthy food was particularly relevant for male athletes living in off-campus accommodation and was also cited as a barrier. This further emphasizes the financial burden associated with healthier eating habits. There is, however, minimal research in the area of the practical challenges athletes can face in achieving optimal nutritional intake. Research is lacking within the sphere of socioeconomic factors influencing dietary choices, cultural influences and accessibility to quality food or supplementation. The study highlights the importance of targeted nutritional education programs to address these barriers. A targeted nutritional educational model could address areas including time-management strategies, nutrition education, hydration practices and basic food preparation skills [18,22,25]. These skills could support student athletes in achieving optimal dietary behaviors. An improvement in nutritional knowledge is likely to have a profound impact on dietary behaviors, training adaptation and thus match performance [25]. Another significant challenge highlighted was the varying levels of nutritional knowledge among players and coaches, with some studies indicating a need for better education and awareness regarding the importance of nutrition in sports performance.

### 3.6. Recommendations to Optimize Nutrition for Enhanced Performance

Given the level of nutritional knowledge among athletes, many studies have highlighted the need for tailored nutritional intervention programs to provide players with evidence-based nutritional advice and practical food skills [6,14]. Mitchell et al. [13] carried out a study to determine nutrition knowledge in elite and non-elite players, and findings indicated that there was little to no difference between the respondent’s nutritional knowledge based on playing level. They further reinforced the importance of nutritional education at all levels of competition. Females were however under-represented in this sample; conversely, a study by Werner et al. [16] showed participants (>50% female), in general, having poor nutritional knowledge with females scoring higher on nutritional knowledge, significantly better than male counterparts. In turn, the contemporary literature in this domain has indicated a deficit in nutritional knowledge, and there persists a need for an innovative nutritional educational model that targets this cohort [4].

## 4. Conclusions

To conclude, this scoping review conducted a comprehensive analysis of the contemporary literature in relation to nutritional knowledge and dietary intake of athletes and described the current gaps in nutritional knowledge and awareness of athletes. Notably, the literature within this field is limited, and there is a need for further research in addressing the nutritional deficits of athletes within different or specific sporting disciplines to enhance performance. Throughout this review, recommendations were identified. This review has highlighted a gap in research specifically addressing nutritional knowledge and dietary intake of athletes and has highlighted the need for the development of a targeted, evidence-based nutritional educational model. This only further elevates the necessity for further research in the area and the necessity to develop further evidence-based nutritional strategies and guidelines which are tailored to the unique demands of athletes in achieving optimum performance. It was noted that there was a further requirement for a broader assessment for procedural knowledge and dietary intake to better understand the practical application of nutritional knowledge [13]. Addressing these gaps will be crucial for helping athletes, in their respective discipline, achieve their performance goals. This scoping review is, however, not without its limitations. Articles included in this review are inclusive of studies of varied methodological designs, and this can make it challenging to draw clear comparisons or conclusions across studies and eliminate casual inferences. There is also limited synthesis of the results which makes it more difficult to provide in-depth insights as the focus is shifted to mapping, charting, and presenting the data. Further studies could examine more specific strategies that could be used to facilitate a tailored nutritional educational program for athletes to maximize impact and efficacy. There were challenges posed with this review regarding the synthesizing of data and identifying clear themes. This was due to the heterogeneity of the studies across the diverse inclusion population. This scoping review has highlighted opportunities to explore understudied topics. These topics include the role of micronutrient intake and sociocultural influences on athletes’ dietary behaviors. Methodological gaps in existing studies were also identified. Varied methodological rigor also influenced the reliability of the conclusions stated. This further emphasizes the need for more research within this field to standardize the evidence base in this field. There is an oversaturation of self-reported data and a lack of longitudinal studies to evaluate the long-term outcomes of nutritional education interventions. By addressing these issues in future research, not only will the evidence base be enhanced but more robust and actionable insights for athletes, coaches, and nutritionists will be highlighted.

## Figures and Tables

**Table 1:** Search terms derived from each concept.

Concept	Search Terms
Nutritional Knowledge of Athletes	“Nutritional Knowledge of athletes” OR “Athletes Dietary Knowledge” OR “Athletes Dietary Intake”
Sports Nutrition	“Sports nutrition” OR “Dietary Requirements of athletes”
	AND
Nutrition Education	“Food Literacy in Sport” OR “Sports Nutrition Education Programs” OR “Athlethe Nutritioanl Training”
Performance and Recovery	“Sports Performance” OR “Athletic Recovery OR “Optimal Performance”

**Table 2:** Charting of eligible included articles for review for synthesis of results.

Author(s) and Year	Country	Objective	Study Design	Sample Size and Population	Results	Conclusion
Renard et al. (2023) [11]	Ireland	To evaluate the cooking and food skills confidence of team sport athletes and to explore the relationship between cooking/food skills confidence and athletes’ demographic characteristics.	A cross-sectional study that collected data via an online questionnaire using Survey Monkey	266 (male: 150, female: 116, age: 24.8 ± 6.1 years)	Females reported greater efficacy and confidence in practical food skills	Male team sport athletes may benefit the most from educational interventions designed to increase cooking and food skills confidence. Lim: Self-reported confidence in cooking and food skills may not be truly representative of ability, and practical measures of cooking and food skills should be developed for future research.
Murphy, John, O’Reilly, James (2020) [12]	Ireland	To investigate the association between sports nutrition knowledge and dietary quality in a sample of adult Irish male hurling players	Sports Nutrition Knowledge Questionnaire (SNKQ) and Australian Recommended Food Score (ARFS) calculated from food frequency questionnaire data. Analysis of variance and linear to assess associations between variables.	265 Participants (129 elite, 136 sub-elite) players	No significant difference in nutrition knowledge (SNKQ) between groups. A significant difference (*p* = 0.02; d = 0.39 ± 0.25; small) in food score (ARFS) between groups	Nutritional Knowledge contributes to dietary quality. Future interventions should focus on specific gaps in knowledge, e.g., energy/carbohydrate requirements.
Mitchell et al. (2022) [13]	Ireland	To examine nutrition knowledge in elite and non-elite Gaelic footballers	Online Survey	90 participants (15.3% female)	No differences between elite and non-elite athletes in nutrition knowledge identified. Athletes with previous nutrition education scored higher than those without	Importance of nutrition education at all levels of athletic competition to improve nutrition knowledge. Lim: elite and women athletes were under-represented
Renard et al. (2022) [11]	Ireland	To examine players knowledge and compared results by playing level, education level, and history of nutrition education	Online Survey	Male Gaelic football players (*n* = 152, mean age = 24.5 ± 5.9). 68 club (sub-elite) and 84 inter-county players (elite)	There are no differences between playing level, when grouped by education level those with master’s degree scored higher in comparison to leaving certificate) Those with previous nutrition education demonstrated higher scores.	Gaelic football players may benefit from evidence-based nutrition education interventions.
Ó Catháin et al. (2020) [14])	Ireland	To examine the dietary intake of Gaelic football player’s 2 days prior to competition, on game day, and for 2 days post competition to assess if (1) player’s intake is in line with current sports nutrition recommendations; (2) if intake varies from day to day, and; (3) if playing level (Elite vs. Sub-elite) influences intake.	A 5-day paper-based food diary	45 players (25 elite and 20 sub-elite).	Playing level had no effect on energy, carbohydrates, or fat intake. Elite players consumed 24% more protein than sub-elite. Regardless of level players consumed inadequate amounts of carbohydrate to support optimal performance and recovery and consumed protein and fat in line with general sport nutrition guidelines	It is necessary to design and implement Gaelic football specific nutrition education-based interventions.
Lydon et al. (2023) [15]	Ireland	To evaluate the effectiveness of a practical food skills and nutrition education workshop and investigate the attitudes towards, and knowledge of, nutrition among young amateur Gaelic Athletic Association (GAA) players.	Quantitative study using a pre-/post-intervention study design	GAA players (*n* = 336) Male and Female	The workshop was effective in improving culinary knowledge, skills, and confidence	Results indicate the benefits of an integrated nutrition education and food skills workshop in improving players’ knowledge of nutrition and increasing confidence in relation to food preparation and cooking. As gender differences were found across a range of key knowledge and confidence outcomes, future food and nutrition education programs may need to be tailored to take account of this
McCrink et al. (2021) [6]	Ireland	To assess the dietary intake, nutrition knowledge and hydration status of Irish Gaelic footballers.	4-day semi-quantitative food record, with the application of Goldberg cut-offs to define acceptable reporters	168 male club/county level Irish Gaelic footballers (median [IQR]; age 23 years	Dietary analysis indicated an energy deficit at the group level. Nutrition knowledge was deemed poor; pre-exercise hydration status denotes dehydration	Irish Gaelic footballers have suboptimal dietary practices and lack nutrition knowledge. Individualized nutrition support may benefit these athletes to meet their nutritional requirements.
Werner et al. (2022) [16]	USA	Assess nutrition knowledge of Division I college athletes.	Survey	128 student-athletes (*n* = 70 female) from eight sporting disciplines	Females scored significantly better than males;	Athletes have low nutrition knowledge
					data	data
Trackman et al. (2018) [5]	Australia	To assess and compare the sports NK of elite and non-elite Australian football (AF) athletes	Validated questionnaire	Elite AF players (*n* = 46) and non-elite AF players (*n* = 53)	knowledge of macronutrients, weight management, and alcohol was better than knowledge of supplements, micronutrients, and sports nutrition. Non-elite athletes achieved higher scores on the questionnaire subsections testing weight management micronutrients and alcohol Overall NK was poor, scores varied among individuals	Professionals working with athletes should undertake an assessment of the athletes’ NK so that they can provide targeted education prog
Jessri et al. (2010) [17]	Iran	To assess the nutrition knowledge and the factors determining this knowledge in Iranian college basketball and football athletes.	Cross-sectional Study	66 basketball and 141 football players from 4 medical and 8 nonmedical universities (male and female)	In both genders, the highest score was obtained for the nutrients; supplements category was poorly answered. In comparison to their peers, a significantly higher score was obtained by women athletes at medical universities and those obtaining nutrition information from reputable sources	Athletes would benefit from nutrition-related training and education.
Magee, Pamela Jane, Gallagher, Alison M., McCormack, Jacqueline M. (2017) [18]	Ireland	To assess the hydration status of university/club level athletes from a range of sports immediately before and after training/competition and to assess their nutritional knowledge.	Urine specific gravity (USG) was measured immediately before and after exercise and BW loss during exercise was assessed. Nutritional knowledge was assessed using a validated questionnaire.	430 Participants	Nutritional knowledge was poor among participants. Dehydration was particularly prevalent among karateka, female netball players, army officer cadets, and golfers	Implications for the education of athletes in relation to their individual fluid requirements around exercise.
Rossi et al. (2017) [19]	USA	To investigate the effects of a sport nutrition education intervention (SNEI) on dietary intake, knowledge, body composition, and performance in NCAA Division I baseball players	Quasi-experimental design to evaluate the effects of a Sport Nutrition Education Intervention	30 NCAA Division I baseball players	Significantly improved sport nutrition knowledge and reduced fat mass in the intervention group	Sport Nutrition Education Intervention proved effective improving nutrition knowledge, enhancing fat mass reduction, and boosting specific performance.
Brauman, Kyle, Achen, Rebecca, Barnes, Jennifer L. (2023) [20]	USA	To reveal what student-athletes believe are the most significant barriers to consuming a healthy diet	survey	169 student-athletes	Key barriers indicate included: lack of time, easy access to unhealthy foods, cost of healthy foods, lack of knowledge about what foods are healthy, and lack of knowledge and skills to cook healthy foods.	Findings from this study could inform future interventions while also presenting initial, usable information for professionals.
Parks et al. (2018) [21]	USA	To describe nutritional behaviors of collegiate athletes and explore practical ways that athletic trainers can collaborate with sports dietitians within a performance nutrition program.	Online survey	(*n* = 682) and 98% (*n* = 678) for 2015 and 2016	Athletic trainers were the most reported source of nutrition information. Lack of time was the primary barrier to eating; Athletes had difficulty following their preferred diet when traveling.	Collegiate athletes face many nutrition challenges and receive nutrition information from various sources. Collaboration between the athletic trainer and sports dietitian is paramount to helping athletes put evidence-based nutrition recommendations into practice.
Arazi, Hamid, Hosseini, Rastegar (2012) [22]	Iran	To compare nutritional knowledge and food habits in collegiate s and non-collegiate athletes.	Survey questionnaire	130 collegiate and non-collegiate male athletes and 120 Collegiate and non-collegiate female athletes	Nutritional knowledge of Iranian non-collegiate athletes was lower than collegiate athletes.	Nutrition educational programs/lessons are one of the solutions to increase knowledge about nutrition.
Ali et al. (2015) [3]	Pakistan	To assess the nutritional knowledge, dietary habits, nutrient intake, and nutritional status of Sultan Qaboos University student athletes.	A cross-sectional study design	71 (49 male and 22 female) student athletes with a mean age of 21.0 ± 1.81 and 19.32 ± 0.72 years (about 8 and a half months)	Significant differences were observed in the sources of nutrition information used. Males gained nutrition information from friends as compared to females from family members Significant differences were also observed in nutritional knowledge and dietary habits scores of male and female athletes. Male had better nutritional knowledge and dietary habits, in comparison to females.	Findings indicate a need for developing strategies in counseling and teaching of athletes to improve their athletic performance and health promotion.
Vázquez-Espino, K.,Rodas-Font, G. and Farran-Codina, A. (2022) [23]	Spain	To assess the NK of athletes from the Fútbol Club Barcelona; and to study its association with self-perceived level of NK, attitude towards nutrition, sources of information, and some dietary habits.	cross-sectional study	Elite athletes (*n* = 264) and compared it to the NK of technical teams of different sports (*n* = 59) and non-athletes (*n* = 183)	Elite athletes had low nutrition knowledge (NK), significantly lower than sports technical teams and nutrition students, and that NK was positively associated with self-perceived NK and healthy dietary habits	Findings indicate the need for more targeted nutrition education plans involving athletes, technical teams, and families.
			data		data	data
Sánchez-Oliver et al. (2020) [24]	Spain	To analyze the pattern of dietary supplements (DS) consumption on federated rugby players, including the analysis of differences based on the sex and competitive level (professional vs. amateur)	specific questionnaire	144 rugby players (83 men and 61 women), of whom 69 were professionals and 75 amateurs	65.3% of participants consumed dietary supplements, with higher prevalence in males and professional; differences in consumption patterns were observed between professional and amateur players in terms of timing, types of DS and purchase sites.	The main reason for DS consumption is for enhancing sports performance
Devlin, Brooke L., Belski, Regina (2015) [2]	Australia	To gain insight into the current level of general and sports nutrition knowledge in elite male AF athletes.	Questionnaire	46 elite male AF players (23.5 ± 2.8 years)	moderate nutrition knowledge was recorded. Findings indicated strengths in broad nutrition recommendations but notable gaps in identifying specific food sources.	Findings indicate a need for targeted nutrition education interventions to address these deficits in knowledge.
Renard, Michèle et al. (2020) [25]	Ireland	To evaluate the nutrition knowledge of female Gaelic games players, compare knowledge by players’ characteristics and identify players’ preferences for information and support.	Validated 35-item questionnaire	28 female Gaelic games players (Age: 23.7 ± 5.0 years)	Elite players scored greater than sub-elite players. Players with higher levels of general education, history of formal nutrition education and previous advice from a nutritionist also presented greater nutrition knowledge	Findings indicate that future education interventions with female Gaelic games players may lead to beneficial changes in dietary behavior
Boucherville et al. (2023) [26]	Finland	To assess the nutritional knowledge and its subsections; general and sports nutritional knowledge of recreational athletes.	A validated, translated, and adapted 35-item questionnaire	409 recreational athletes (male: 173, female: 236, age = 32.4 ± 9.6 years	Recreational athletes demonstrated average general nutritional knowledge but poor sports-specific knowledge, with higher scores observed in males, younger participants, and those with prior nutritionist appointments or formal nutrition education.	Findings indicate a lack of nutritional knowledge in recreational athletes, those without an appointment with a registered nutritionist and formal nutritional education.

## Data Availability

The data underlying this article is available in the article.
